# Extracellular Vesicles from Parasitic Helminths Contain Specific Excretory/Secretory Proteins and Are Internalized in Intestinal Host Cells

**DOI:** 10.1371/journal.pone.0045974

**Published:** 2012-09-24

**Authors:** Antonio Marcilla, María Trelis, Alba Cortés, Javier Sotillo, Fernando Cantalapiedra, María Teresa Minguez, María Luz Valero, Manuel Mateo Sánchez del Pino, Carla Muñoz-Antoli, Rafael Toledo, Dolores Bernal

**Affiliations:** 1 Área de Parasitología, Departamento de Biología Celular y Parasitología, Universitat de València, Burjassot, Valencia, Spain; 2 Servicios Centrales de Soporte a la Investigación Experimental (SCSIE) de Universitat de València, Burjassot, Valencia, Spain; 3 Servicio de Proteómica, Centro de Investigación “Príncipe Felipe”, Valencia, Spain; 4 Departamento de Bioquímica y Biología Molecular, Universitat de València, Burjassot, Valencia, Spain; Institut national de la santé et de la recherche médicale - Institut Cochin, France

## Abstract

The study of host-parasite interactions has increased considerably in the last decades, with many studies focusing on the identification of parasite molecules (i.e. surface or excretory/secretory proteins (ESP)) as potential targets for new specific treatments and/or diagnostic tools. In parallel, in the last few years there have been significant advances in the field of extracellular vesicles research. Among these vesicles, exosomes of endocytic origin, with a characteristic size ranging from 30–100 nm, carry several atypical secreted proteins in different organisms, including parasitic protozoa. Here, we present experimental evidence for the existence of exosome-like vesicles in parasitic helminths, specifically the trematodes *Echinostoma caproni* and *Fasciola hepatica*. These microvesicles are actively released by the parasites and are taken up by host cells. Trematode extracellular vesicles contain most of the proteins previously identified as components of ESP, as confirmed by proteomic, immunogold labeling and electron microscopy studies. In addition to parasitic proteins, we also identify host proteins in these structures. The existence of extracellular vesicles explains the secretion of atypical proteins in trematodes, and the demonstration of their uptake by host cells suggests an important role for these structures in host-parasite communication, as described for other infectious agents.

## Introduction

Helminth infections are among the most neglected tropical diseases [1]. Many species of helminths are parasitic multicellular organisms of medical and economic importance, as they infect humans and sometimes induce fatal diseases. Moreover, parasitic helminth infections in livestock are responsible for significant economic losses due to decreases in animal productivity and the cost of antihelminthic treatments of parasitized individuals [1]. Echinostomes are intestinal trematodes with no tissue phases in the definitive host. They are important parasitic flatworms invading domestic and wildlife animals and occasionally humans [2].

These flukes, particularly *Echinostoma caproni* (Trematoda: Echinostomatidae) have been used for years as experimental models in different areas of parasitology because they are able to parasitize a wide range of invertebrate and vertebrate hosts during their life cycle [3]. More specifically, our group has used *E. caproni* as a model for chronic and acute intestinal helminth infections depending on the rodent host used [3].

*Fasciola hepatica* is a parasite of the liver and bile ducts of human and non-human definitive hosts, such as livestock [2], [4]. The definitive host, including humans, becomes infected after ingestion of contaminated vegetation. The parasite excysts in the small intestine and juvenile worms penetrate through the gut wall and enter the peritoneal cavity. After 10–12 weeks of tissue migration the parasites enter the bile ducts where they mature [5]. Although fascioliasis has been traditionally considered as a livestock disease, it is now recognized as an important emerging zoonotic human disease. It is estimated that between 2.4 and 17 million people are currently infected and 91 million are at risk of infection [2].

Although studies of host-parasite interactions have led to important discoveries related to the identification of potential new targets for diagnosis and treatment, as well as new vaccine targets for helminthiases [1], [2], further studies are required to identify new and specific targets for effective control of these important diseases. In this context, interesting helminth target molecules consist mainly of those present at the external surface (cuticle in nematodes and tegument in trematodes) and the excretory/secretory products (ESP) [6]. We and others have studied the proteins present in ESP from different helminths, which exhibit a common pattern in all the species studied, where cytoskeletal proteins, nuclear proteins and glycolytic enzymes are the most abundant [2], [6], [7]. Recent studies have indicated that the secretomes of many helminth pathogens contain a variety of highly-abundant proteins that are homologs of damage-associated host molecules. Helminths could have evolved mechanisms similar to their host in order to prevent their elimination by humoral and cellular immune responses [8].

In multicellular organisms, cells communicate with each other via extracellular molecules, but also by releasing membrane vesicles into their extracellular environment that can affect the cells that encounter these structures in complex ways. When, in 1963, L.T. Threadgold first characterized the tegument of *F. hepatica*, he described a great number of small membrane-limited vesicles, possibly indicating the process whereby metabolites may be secreted [9]. Moreover, Andresen *et al.* (1989) also reported the existence of “membrane bound vesicles” in *E. caproni* [10]. Nevertheless, the research on secretion vesicles and their wider involvement in intra and extracellular signaling began to emerge in the last decade [11]. This interest is reflected by the increasing number of papers published in last years, and the increasing information provided in databases like Exocarta (http://exocarta.org) [12]. For recent reviews see [13]–[16].

Exosomes represent a specific subtype of secreted vesicles. They were originally identified as small (30–100 nm) membrane vesicles that are actively secreted by several types of cells. It has been demonstrated that exosomes contribute to functions such as tissue repair, neural communication, immunological response and the transfer of pathogenic proteins [11], [14], [16]. It has been shown that protozoan parasites also secrete exosomes, representing a broad-based mechanism of protein export which can contribute either to tolerance of an invader (by dampening immune response), or, conversely, to elicit pathologic inflammatory reactions in the host [17], [18]. In contrast, although several approaches have been employed to study the biology of helminths and their interactions with the host, the secretion of these structures by parasitic helminths has been poorly studied. The demonstration of the existence of exosome-like vesicles in helminths may offer a new point of view for the study of helminth infections. In the present study, we demonstrate the secretion of exosome-like vesicles and their potential role in the communication between the parasites and the host in two trematodes, *Echinostoma caproni* and *Fasciola hepatica*.

## Materials and Methods

### Ethics Statement

This study was performed in strict accordance with the recommendations of the Ethics Committee of Experimental and Animal Welfare of the Universitat de València, which approved the protocols on March, 4^th^, 2009. Our animal protocols followed Spanish regulations (Royal Decree 1201/2005, published in BOE 21-10-2005) which adhere to European regulations (Directives 86/609/CEE and 2003/65/CE). All surgery was performed under sodium pentobarbital anesthesia, and every effort was made to minimize suffering.

### Parasites

*E. caproni* adults were obtained from ICR mice (*Mus musculus*) after four weeks of experimental infections with 75 metacercariae/each as previously described [19]. Animals were maintained under standard conditions with food and water ad libitum. The worm egg release was investigated daily in each infected animal, as described previously [20].

*F. hepatica* adults were obtained from cow livers in Mercavalencia S.A. slaughterhouse.

### Excretory–secretory products (ESP)

To prepare ESP, adult worms from either *E. caproni* (collected from the intestines of experimentally-infected mice) or *F. hepatica* (collected from cow livers from local abattoirs) were thoroughly washed with PBS and maintained in RPMI-1640 culture medium containing 100 U penicillin and 100 µg/mL streptomycin (all from Sigma), at concentrations either of 10 worms/mL (*E. caproni*) or 2 worms/mL (*F. hepatica*) at 37°C for 5 h. In all experiments, adult parasites were alive after incubations.

### Extracellular vesicle purification

Exosome-like vesicles were purified by differential centrifugation as suggested by Mathivanan *et al.*, (2010) [13], using a protocol based on ultracentrifugation coupled to membrane filtration to eliminate large contaminating extracellular vesicles, and following validation by electron microscopy, as described for other systems [21], [22]. Briefly, after the incubation period, the parasite culture media was collected and centrifuged at low speed (first at 300 g/10 min, and then at 700 g/30 min) to remove larger debris, and the resulting supernatant was centrifuged at 15,000 g for 45 min at 4°C. ESP supernatants were then filtered using an ultrafiltration membrane (0.2 µm; Schleicher & Schuell) and centrifuged at 120000 g/1 h at 4°C in an Optima TL100 tabletop ultracentrifuge (Beckman) using a TLA-55 rotor.

### Electron microscopy: TEM, Immunochemistry-EM and SEM

For TEM, LR-white resin inclusion was performed fixing parasite adults and extracellular vesicles with glutaraldehyde 2.5%, washed with phosphate buffer 0.1 M pH 7.2, and post-fixed with 2% osmium tetroxide in phosphate buffer. After washes with water, they were sequentially dehydrated in 30% EtOH, 50% EtOH, 70% EtOH and 96% EtOH. Finally, samples were sequentially incubated for 2 h in 33% LR-white resin in 96% EtOH, 66% LR-white resin in 96% EtOH, 66% LR-white resin in 100% EtOH and 100% LR-white resin in 100 EtOH Samples were filtered in resin and polymerized at 60°C for 48 h. Ultrathin slides (60 nm) were finally stained with 2% uranil acetate prior to viewing by transmission EM (TEM) using a Jeol JEM1010 microscope at 60 kV. Images were acquired with a digital camera MegaView III with Olympus Image Analysis Software.

For the immuno-gold labeling assays with antibodies, purified exosome-like vesicles and *E.caproni* adults were fixed with Karnovsky's fixative and then processed in resin as previously described. Grids containing the samples were blocked with PBS/0.8% BSA/0.1% gelatin, and 2 µL of each antibody in PBS/0.5% BSA were added. Goat anti-enolase antibody (Santa Cruz Biotechnology); rabbit sera obtained against actin from *Echinostoma caproni* and rabbit sera obtained against Leucine aminopeptidase (LAP) from *Fasciola hepatica* [23] (kindly provided by Dr. C. Carmona, Universidad de la Republica, Montevideo, Uruguay) were used at dilution of 1/20. The grids were then washed with PBS/0.5% BSA, incubated with gold-labeled secondary antibodies (Donkey anti-goat coupled to 18 nm gold particles; donkey anti-rabbit coupled to 12 nm gold particles, all from Jackson Immunoresearch) in PBS/0.5% BSA for 30 min, and then washed in 100 µL drops of PBS/0.5% BSA. Control grids incubated with only secondary antibodies were also used. The grids were stained with 2% uranyl acetate and then viewed for TEM using a Jeol JEM1010 microscope at 60 kV and images were acquired with a digital camera MegaView III with Olympus Image Analysis Software.

For SEM adult worms were fixed in Karnovsky's fixative, washed in buffer and post-fixed for 1–2 h with 2% osmium tetroxide in 0.1 M sodium phosphate buffer pH 7.2, and dehydrated by critical point. The mounted specimens were coated with Gold/Paladium and examined in a Hitachi S4100 scanning electron microscope at 5 kV.

### Exosome-like vesicles membrane staining

Extracellular vesicles isolated as described before were labeled with FM4–64 stain (Molecular Probes Inc.) according to the manufacturer's protocol. Briefly, purified vesicles from 6 mL of ESP were resuspended in 400 µL of PBS and mixed gently with FM4–64 (added to a final concentration of 5 µM) for 10 min on ice. The samples were then washed by ultracentrifugation at 120,000 g to remove the excess dye and resuspended in 200 µL of PBS. Labeling of vesicles was confirmed by confocal light microscopy in an Olympus FV1000 microscope. Optical excitation was carried out with a 488 nm argon laser beam and fluorescence emission was detected in the range of 655–698 nm.

### Culture assays with labeled exosome-like vesicles and confocal microscopy

Rat intestinal epithelial cancer (IEC-18) cells (ATCC n° CRL-1589, kindly provided by Dr. Duñach, Universitat Autonoma de Barcelona, Spain) were routinely grown in Dulbecco's modified Eagle's medium supplemented with 10% fetal calf serum until 80% confluence. IEC-18 cells were grown in 800 µL of DMEM media in µ-slide chambers (ibiTreat, Ibidi GmbH) at 37°C and 5% CO_2_. Prior to assays, cells were starved in DMEM without FBS for 4 h.

We performed duplicate assays with 10 and 100 µL of FM4–64 labeled vesicles per well, and keeping the cultured cells in a chamber at 37°C coupled to an Olympus FV1000 laser scanning confocal microscope for the times indicated.

As a control, IEC-18 cells were grown in several µ-slide chambers, starved in DMEM without FBS for 4 h, cooled to 4°C and incubated with 10 and100 µL of FM4–64 labeled vesicles per well, keeping the cultured cells at 4°C. Images were recorded at 0, 30, 60, 90 and 120 minutes.

Images were obtained using the Olympus FV10-ASW software v 02.01.03.10 (Olympus Corporation) and Olympus UPLSAPO 60× oil objective. Optical excitation was carried out with a 488 nm argon laser beam and fluorescence emission was detected in the range of 655–698 nm.

### Proteomic analysis

Purified extracellular vesicles were precipitated with 100 µL of CHCl_3_/MeOH. The pellet was dissolved in 50 µL of 50%Trifluoroethanol (TFE), 50 mM Ammonium bicarbonate (ABC). Then, the samples were sequentially treated with 2 mM DTT (60°C/30 min), 5 mM Iodoacetamide (IAM) (room temperature in the dark/30 min), and 10 mM DTT (room temperature/30 min). After dilution of TFE (<5%) with 50 mM ABC, samples were treated with 500 ng trypsin (Promega) (37°C/overnight). Digestions were stopped with 50 µL of Trifluoroacetic acid (TFA) (Sigma) 10% (final pH 1), and dried in a vacuum centrifuge. The final mixtures were reconstituted with 10 µL of 0.1% TFA before analyzing by LC-MS/MS, as previously described [24].

Five microliters of the resulting suspension were delivered to a trap column (LC Packings Amsterdam, C18 PepMap100, 5 µm; 300 µm×5 mm) using capillary HPLC (Switchos, LC Packings) via an isocratic flow of mobile phase (0.1% TFA in water) at a rate of 30 µL/min for 3 min. The flow rate was then switched to 300 nL/min, and the peptides were flushed into the analytical column (LC Packings C18 PepMap100, 3 µm, 100 Å, 75 µm×15 cm) and eluted via a mobile phase gradient: 2–30% of B buffer in A buffer (A buffer: 0.1% formic acid in water, B buffer: 0.1% formic acid in 95% acetonitrile) over 120 min. The elution was directly applied to a nanospray source of a QSTAR XL instrument (Applied Biosystems). The QSTAR XL was operated in information-dependent acquisition mode, in which a 1-s TOF MS scan from 400–2000 m/z was performed, followed by 3-s product ion scans from 65–2000 m/z on the three most intense doubly or triply charged ions.

### Database search

Database search on NCBInr databases was performed using both the MASCOT (Matrix-Science) search engine and the ProteinPilot software v3.0.1 (Applied Biosystems) for metazoan organisms, as previously described [24]. Basically, MASCOT searches allowed one missed cleavage and a tolerance on the mass measurement of 100 ppm in MS mode and 0.5 Da for MS/MS ions. We used carbamidomethylation of Cys as a fixed modification and oxidation of Met and deamidation of Asn and Gln as variable modifications [24]. With the ProteinPilot software, the default parameters for Paragon algorithm [25] were used.

## Results

### The parasitic trematodes *Echinostoma caproni* and *Fasciola hepatica* produce exosome-like vesicles

**Figure 1:**
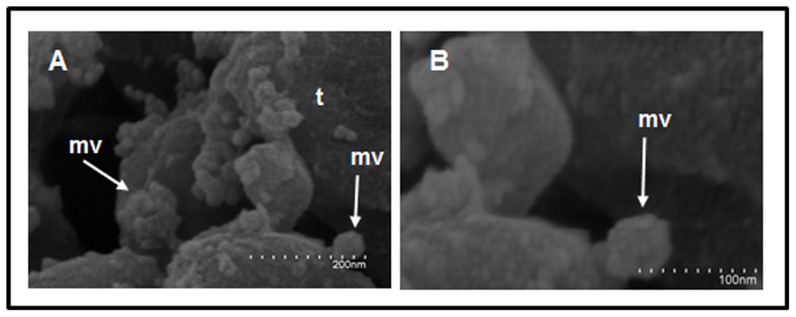
Microvesicles are present at the surface of the *Echinostoma caproni* tegument. Parasite tegumental area as seen by scanning electron microscopy (SEM) at different magnifications: ×200000 (A), and ×350000 (B). t: tegument; mv: microvesicles. The dots in the scale bars correspond to 1/10 of the length indicated in the figure.

Scanning electron microscopy (SEM) assays with *Echinostoma caproni* adults showed the presence of microvesicles on the surface of their tegument. The external surface of *E. caproni* adults is evaginated into vesicular-shaped bodies with a size ranging from 30–100 nm, which is in the range of exosome-like structures (Fig. 1A, B).

**Figure 2:**
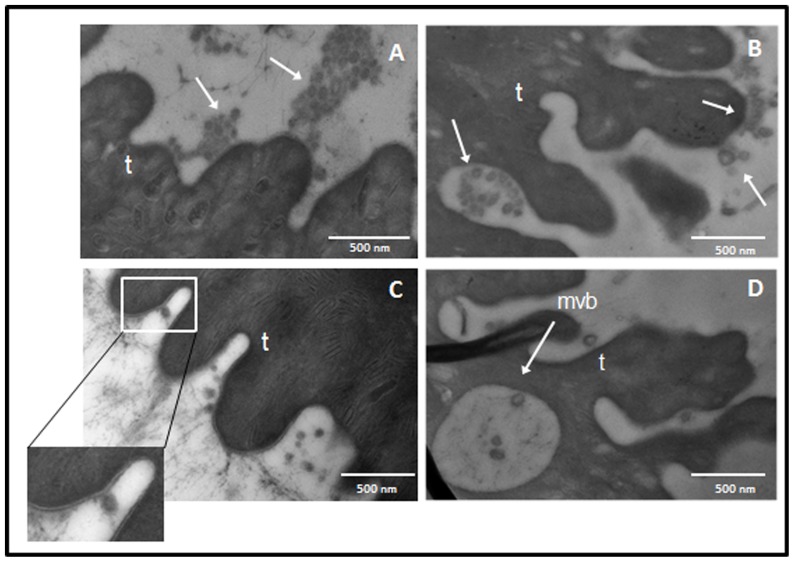
*Echinostoma caproni* secretes exosome-like vesicles. Production of *E. caproni* vesicles seen by transmission electron microscopy (TEM) at different magnifications: ×100000 (A), ×200000 (C), ×80000 (B, D). t: tegument; mvb; multivesicular bodies.

Transmission electron microscopy (TEM) assays confirmed the presence of different microvesicular structures on the *E. caproni* adult tegument (Fig. 2). They appeared as typical spherical structures released into the media (Fig. 2A, B, C), as previously described in *E. caproni* and *Fasciola hepatica* [9], [10]. These vesicles are apparently nipped off into the environment. We also detected these vesicles in membranous multivesicular bodies similar to exosome structures (Fig. 2 D).

**Figure 3:**
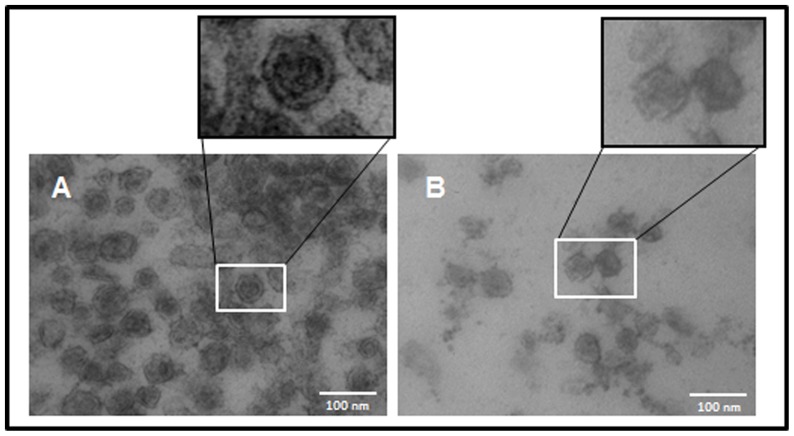
Exosome-like vesicles obtained from *Echinostoma caproni* and *Fasciola hepatica*. Excretory/secretory materials (ESP) from *E. caproni* (A) and *F. hepatica* (B) were ultracentrifuged and the insoluble material was analyzed by transmission electron microscopy. Membranous vesicles of 30–100 nm of diameter are observed. Magnification ×200000.

To ascertain whether these exosome-like vesicles were also produced by other trematodes and to explore their nature, we next carried out classical purification assays of these structures from *E. caproni* and *F. hepatica* ESPs, and used TEM to visualize them. As shown in figure 3, abundant round-shaped material with the expected size of exosomes was obtained after ultracentrifugation from both *E. caproni* (Fig. 3A) and *F. hepatica* (Fig. 3B) ESPs, confirming their presence in the excreted/secreted material by both parasitic trematodes.

### Helminth parasite exosome-like vesicles contain typical excretory/secretory proteins

We next carried out two complementary approaches (proteomics and immune-TEM) to identify the proteins present in purified extracellular vesicles from *E. caproni* and *F. hepatica*.

Exosome-like vesicle preparations, which were previously confirmed by TEM, were subjected to proteomic analysis. Purified vesicles were digested with trypsin and no proteins were detected by MS/MS (data not shown). In order to release the protein content, purified vesicles were precipitated with CHCl_3_/MeOH and then digested with trypsin. Protein identification using MASCOT from three different experiments confirmed the presence of 45 different proteins in exosome-like vesicles from *E. caproni* ESP (Table S1). Application of the ProteinPilot software allowed for the identification of 6 additional proteins (Table S2). Most of the identified proteins had been found in ESP in previous studies, including cytoskeletal proteins (i.e. actin, tubulin, myosin, paramyosin, tropomyosin), glycolytic enzymes (i.e. enolase, aldolase, GAPDH, PEPCK), calcium-binding proteins (i.e. calmodulin, calponin), nuclear proteins (histones and elongation factors), as well as stress-related proteins (i.e. HSPs) and detoxifying enzymes like peroxiredoxins [2], [19], [26]. In addition to parasite proteins, the vesicles from *E. caproni* adults also contained host-derived proteins. In *E. caproni* exosome-like vesicles, MASCOT searches produced the identification of 36 different host proteins in these vesicles (Table S1). The identified mice proteins mainly corresponded to immunoglobulins, histones, partial sequences of mucins and metabolic enzymes (Table S1).

The exosome proteome of *E. caproni* accounted for 54% (Table S3) of the proteins currently identified in the secretome [19], [27].

In the case of *F. hepatica* vesicles, we could identify 79 different proteins (Table S4). *F. hepatica* exosome-like vesicles exhibited qualitative differences in composition, when compared to *E. caproni* vesicles, including a higher number of proteases (like cathepsins and leucine aminopeptidase, LAP), as well as detoxifying enzymes (i.e. heat-shock proteins, and fatty-acid binding proteins), all of them also previously described as being present in *F. hepatica* ESP [6], [28]–[30]. The proteins identified in these vesicles accounted for the 52% of the secretome (Table S5).

The high protein overlap between the proteins in the secretome and the exosomes suggests that these vesicles are the primary mechanism of protein release from these trematodes similarly to what has been described for *Leishmania* [31].

In *F. hepatica* vesicles, we identified 19 host proteins including immunoglobulins, metabolic enzymes and typical exosomal molecules like CD19 (Table S4).

**Figure 4:**
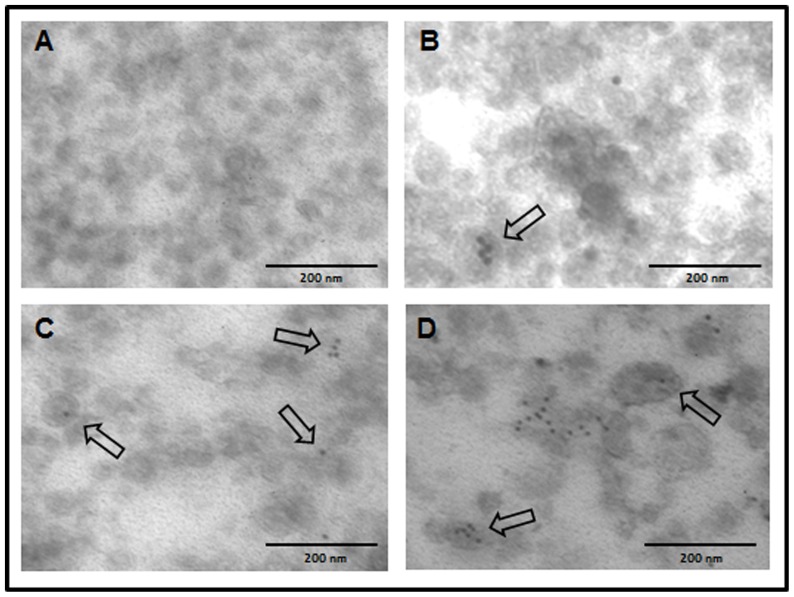
Trematode exosomes-like vesicles contain typical ESP proteins. *E. caproni* vesicles probed with preimmune sera (A). Immunodetection of enolase (B), actin (C) in *E. caproni* exosomes, and leucine aminopeptidase (LAP) in *F. hepatica* exosomes (D) are shown. Magnification ×100000.

We further confirmed the presence of some of the most abundant proteins (actin, enolase, and LAP) in purified vesicles by immunohistochemistry and TEM using antibodies against some of the abundant molecules [26], [28]. As shown in figure 4, gold-labeling was detected in *E. caproni* vesicles when using antibodies specific for enolase (Fig. 4B) and actin (Fig. 4C), and no label was detected when incubating with pre-immune antibodies, confirming the specific detection of these molecules in the exosome-like vesicles (Fig. 4A). Gold-labeled antibodies against *F. hepatica* LAP [23] clearly detected the presence of this protease in *F. hepatica* vesicles (Fig. 4D), confirming its identification by proteomics. No label was detected when *F. hepatica* exosome-like vesicles were incubated with mock antibodies (data not shown).

**Figure 5:**
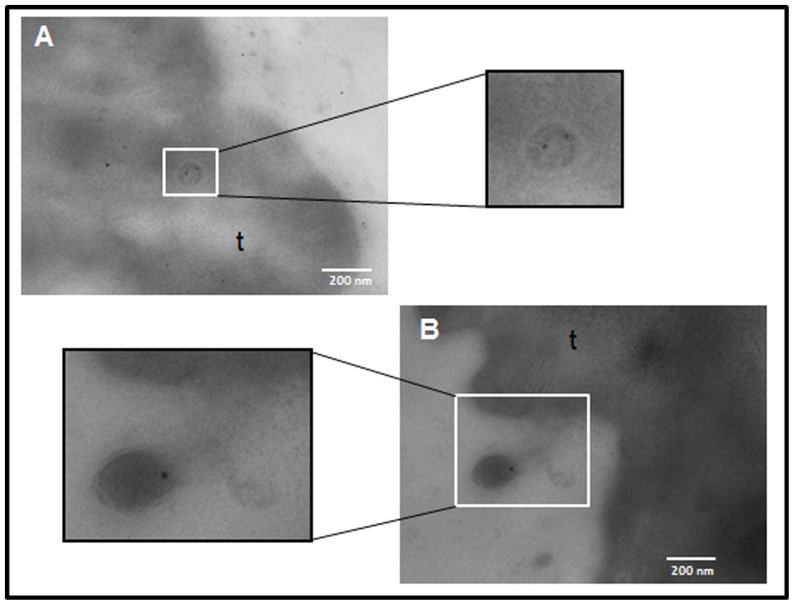
*E.caproni* tegument presents microvesicles containing gold labeled actin. t: tegument. Magnification ×80000.

To investigate the origin of these vesicles, we next performed immunohistochemistry and TEM of *E. caproni* and *F. hepatica* adults with antibodies against actin and LAP, respectively (Fig. 5).These assays revealed gold labeled vesicles in *E. caproni* tegument suggesting the process whereby actin molecules may be secreted (Fig. 5A, B). Labeling was detected in vesicles that were detached from the surface (Fig. 5B). Similar results were obtained in *F. hepatica* tegument when using anti-LAP antibodies (data not shown). We conclude that exosome-like vesicles are already formed in the tegument of these trematodes.

### *Echinostoma caproni* exosome-like vesicles are internalized by rat intestinal epithelial cells in culture

**Figure 6:**
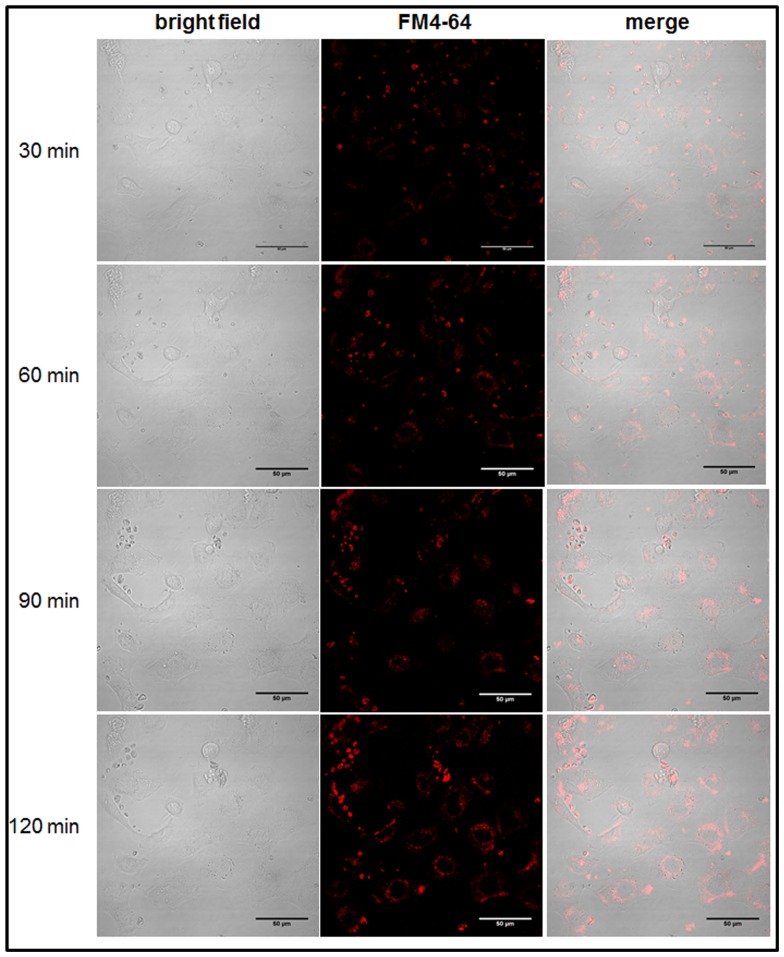
Uptake of *E. caproni* exosomes by intestinal IEC-18 cells. Confocal images of FM4–64 stained *E. caproni* exosomes (red) after different times of incubation. Magnification ×600. The scale bars correspond to 50 mm.

Once confirmed the existence and composition of *E. caproni* exosome-like vesicles, we next performed functional assays to ascertain their role in host-parasite interactions. We analyzed whether these structures could interact with host cells and be internalized in *cultured cells*. Intestinal rat cell (IEC-18) cultures were incubated with FM4–64-labeled *E. caproni* vesicles for 2 h. Images were taken at the indicated time points by time-lapse confocal fluorescence microscopy (Fig. 6). Upon addition of labeled vesicles to the culture media at 37°C, fluorescence was observed outside the cells (data not shown). As observed in figure 6, after only 30 min, fluorescence was observed at the cell surface and the intracellular fluorescence increased markedly during the time course. At 4°C, the fluorescence was observed outside the cells at the different times analyzed (data not shown). These results strongly suggest that the parasite extracellular vesicles are taken up by the rat cells in a time-dependent and metabolically-dependent manner, most likely by endocytosis, confirming interspecific communications mediated by exocytic vesicles.

## Discussion

In this study, we report the active release of microvesicles exhibiting the morphological and biochemical characteristics of typical exosomes by the parasitic trematodes *E. caproni* and *F. hepatica*. The present study constitutes the first report of the existence and composition of exosome-like vesicles in parasitic helminths, since functional exosomes in helminths only had been previously described in the free-living nematode *Caenorhabditis elegans* [32]. Furthermore, our results indicate that these vesicles may play an essential role in host-parasite communication since they are actively taken up by intestinal rat cells. Our results confirm the ultrastructure of the tegument of *F. hepatica* described by Threadgold [9] in which describes “a great number of small membrane-limited vacuoles and vesicles”. Interestingly, Andresen *et al.* [10] also reported the existence of “membrane bound vesicles” containing surface antigens in the adult worms of *E. caproni* that may well correspond to the structures described herein (Fig. 2). Remarkably, we found multivesicular bodies near the surface containing typical spherical exosome-like vesicles (Fig. 2D), exhibiting the typical morphology of exosome vesicles [13]–[16].

Most of the proteins we identified in the extracellular vesicles correspond to those described previously in the secretome of *E. caproni*, *F. hepatica* and other parasitic trematodes [2], [19], [29], [30]. In fact, more than a half of the molecules identified in the secretome of *E. caproni* and *F. hepatica* are found in these exosome-like vesicles. This suggests that extracellular vesicles constitute an important mechanism for protein export in trematodes, as suggested for the parasitic protozoa *Leishmania* [31]. Furthermore, this mechanism may explain the presence of atypical proteins lacking classical secretion signal peptides, like enolase, in the helminth ESP [32]–[38]. A very recent study has confirmed the presence of enolase in the outer membrane vesicles from spirochaetae as responsible for fixing enymes involved in external proteolysis in the pericellular environment, having roles in nutrition and in enhancing dissemination [39]. Future studies should confirm the role of secreted helminth enolases in the host-parasite interaction.

Interestingly, all the molecules identified herein are among the most abundant exosome proteins identified in other organisms (see Exocarta.org; [12]). Among the proteins identified in exosomes, some are considered as damage-associated molecular pattern molecule (DAMP) homologs, which lack typical secretion signals and exit the cell by non-classical secretory pathways [8], [40]. We now confirm that some of these DAMP homologues (i.e. annexins and heat shock proteins) are actively secreted in trematodes. An interesting DAMP homologue found in both *E. caproni* and *F. hepatica* parasites was thioredoxin peroxidase (peroxiredoxin). This molecule is also considered as a pathogen-associated molecular pattern molecule (PAMP) [40], able to modulate immune cells [8], [41], thus constituting a promising target for pharmacological interventions [8].

One of the most striking features in our study is the evidence that extracellular vesicles may play an important role in the communication between the parasite and the host. This is based on: (i) we have shown that parasite vesicle proteins are species-specific; (ii) we have identified some host proteins in the parasite exosome-like vesicles; and (iii) parasite vesicles are incorporated into live intestinal cells. Although some common proteins were identified in the exosome-like vesicles released from *E. caproni* and *F. hepatica*, particularly structural and metabolic enzymes, our results clearly indicate that they are species-specific, since major differences were detected between both trematodes. For example, vesicles released from *F. hepatica* were enriched in stress-related proteins and proteases. The presence of proteases, such as cathepsins and aminopeptidases, in the ESP of *F. hepatica* has been reported previously and implicated in tissue migration of the juvenile worms from the intestine to the definitive microhabitat in the bile ducts [42], [43]. Similarly, in the migrating parasitic protozoa *Trypanosoma brucei*, the presence of cathepsin L-like in 50- to 100 nm vesicles budding from the coated plasma membrane has also been demonstrated [44]. In contrast, in extracellular vesicles of *E. caproni*, which is an intestinal trematode with no tissue phases within the host, no proteases were identified. This suggests that biological characteristics of the parasite species determine the protein composition of the vesicles and their involvement in the success of the infection.

The identification of host proteins in purified parasite exosome-like vesicles clearly implicates these structures in host-parasite communication processes. The host proteins identified also appear to be species-specific, corresponding to those highly produced by hosts in response to helminth infection in each case (i.e. mucin-2 was only identified in *E. caproni* vesicles, whereas CD19 was only found in *F. hepatica* ones). Recent studies have also identified host proteins like IgA heavy chain constant region in secretion preparations of *Fasciola hepatica* [30]. The role of these host proteins in parasitic vesicles warrants further investigation.

Furthermore, we show that labeled parasite extracellular vesicles are incorporated into intestinal cells in culture, suggesting a role for these structures in host-parasite communication. Other examples of the involvement of extracellular vesicles in host-pathogen communication include HIV [45], [46], spirochaetes like *Borrelia burgdorferi* [39], fungi like *Cryptococcus neoformans* [47], [48] or *Malasezia sympodialis* [49], and protozoans like *Trypanosoma brucei* [50] or *Leishmania* spp. [31]. In some cases, exosomes have been implicated in modulating the host response to those pathogens [31], [47], [49]. Studies are needed to confirm this role for exosomes in parasitic helminths, but would support previous studies suggesting an important role for helminth ESP in modulating the host immune response [6].

Recent studies have demonstrated that exosomes can be internalized by endocytosis [51]. We believe it could be also the case for helminth vesicles, which could be internalized either by an unspecific endocytic pathway, with no ligand-receptor involved, or by specific mechanisms requiring unidentified proteins that would act as a ligand for receptor recognition. Currently, we have not identified proteins at the exosome-like vesicle surface, and more studies are required to address this question.

In summary, although the secretion of exosome-like vesicles has been demonstrated in several organisms, we have shown the production of these structures by parasitic helminths for the first time. These structures have been isolated and purified following specific and validated protocols used for a wide range of organisms and systems [13], [17], [21], [22], [44]–[49].

Moreover, we reveal the involvement of these structures in the communication with the host, suggesting an important role for exosome-like vesicles in the establishment of the infection. In this context, further studies will address whether these vesicles constitute good targets for new control strategies in parasitic diseases, implementing new vaccines, diagnostic and treatment tools.

## Supporting Information

Table S1 **Proteins from** ***Echinostoma caproni*** **exosome-like vesicles identified using MASCOT search engine (Matrix-Science).**
(XLSX)

Table S2 **Proteins from** ***Echinostoma caproni*** **exosome-like vesicles identified using ProteinPilot software v2.0.1 (Applied Biosystems).**
(XLSX)

Table S3 **Extracellular vesicles (ECV) account for 56% of the** ***Echinostoma caproni*** **secretome (refs. 19, 27 and 35).**
(XLSX)

Table S4 **Proteins from** ***Fasciola hepatica*** **exosome-like vesicles identified using MASCOT search engine (Matrix Science).**
(XLSX)

Table S5 **Extracellular vesicles (ECV) account for 52% of the** ***Fasciola hepatica*** **secretome (refs. 28, 30, 52).**
(XLSX)
